# Bulked segregant analysis reveals candidate genes responsible for dwarf formation in woody oilseed crop castor bean

**DOI:** 10.1038/s41598-021-85644-1

**Published:** 2021-03-18

**Authors:** Zaiqing Wang, Anmin Yu, Fei Li, Wei Xu, Bing Han, Xiaomao Cheng, Aizhong Liu

**Affiliations:** 1 Department of Economic Plants and Biotechnology, Yunnan Key Laboratory for Wild Plant Resources, Kunming Institute of Botany, Chinese Academy of Sciences, Kunming, 650204 China; 2 Key Laboratory for Forest Resources Conservation and Utilization in the Southwest Mountains of China, Ministry of Education, Southwest Forestry University, Kunming, 650224 China; 3 University of the Chinese Academy of Sciences, Beijing, 100049 China

**Keywords:** Agricultural genetics, Plant breeding

## Abstract

Plant dwarfism is a desirable agronomic trait in non-timber trees, but little is known about the physiological and molecular mechanism underlying dwarfism in woody plants. Castor bean (*Ricinus communis*) is a typical woody oilseed crop. We performed cytological observations within xylem, phloem and cambia tissues, revealing that divergent cell growth in all tissues might play a role in the dwarf phenotype in cultivated castor bean. Based on bulked segregant analyses for a F_2_ population generated from the crossing of a tall and a dwarf accession, we identified two QTLs associated with plant height, covering 325 candidate genes. One of these, *Rc5NG4-1* encoding a putative IAA transport protein localized in the tonoplast was functionally characterized. A non-synonymous SNP (altering the amino acid sequence from Y to C at position 218) differentiated the tall and dwarf plants and we confirmed, through heterologous yeast transformation, that the IAA uptake capacities of *Rc5NG4-1Y* and *Rc5NG4-1C* were significantly different. This study provides insights into the physiological and molecular mechanisms of dwarfing in woody non-timber economically important plants, with potential to aid in the genetic breeding of castor bean and other related crops.

## Introduction

Dwarfism of various crops triggered the Green Revolution allowing agricultural modernization and therefore unprecedentedly increased crop production^1, 2^. For woody non-timber economic plants such as fruit tree and woody oilseed crops, dwarfism is a critical way to breed varieties because dwarfed varieties often not only bring higher yields, but also allow compact planting and convenient management in the field^3, 4^. The genetic basis of plant height is complex, involved in multiple regulatory mechanisms at the physiological and molecular levels.

Usually, auxin is considered a key regulator to control plant height because of its critical roles in regulating cell division, elongation, and differentiation at the meristem such as apical meristem and vascular cambia^5, 6^, and orchestrating plant growth^7–9^. When auxin biosynthesis or its cellular transport is interrupted, apical growth is often repressed, leading to enhanced branch growth and development, resulting in a dwarfed and typically high yielding phenotype^10–12^. Loss-of-function mutations of genes such as *TAA1* (Trytophan aminotransferase of Arabidospsis) and *YUCs* (encoding YUCCA flavin monooxygenase-like enzymes) involved in regulating IAA biosynthesis^7, 13^, and *TIR1* (encoding transport inhibitor response1) and *TMKs* (encoding transmembrane kinase auxin) involved in sensing auxin signals, often cause a low content of IAA and a disruption of the IAA signal network, resulting in a dwarf phenotype in Arabidopsis^14–16^. Intercellular transport of auxin involves various transport proteins. Functional loss of transporters such as Arabidopsis auxin resistant1 (AUX1, a polar imported transporter), PIN-FORMED (PIN) 1–3, 6 and 7 (polar exported transporters), and the ATP-binding cassette subfamily B1 (ABCB1) and 19 (lateral efflux transporters) also lead to a dwarf phenotype or shorter hypocotyl in Arabidopsis and rice^17–23^. The intracellular transport of auxin often maintains or changes intracellular homoeostasis of free IAA in the cytoplasm and storage conjugated IAA in the vacuole or endoplasmic reticulum (ER)^24, 25^. When disfunction of cellular IAA transporters located in ER including PIN5, PIN-LIKE2 (PILS2) and PILS5 give rise to a reduced content of free IAA in cytoplasm, it results in a dwarf phenotype^24, 26^. Functional loss of WAT1 (Walls are thin 1 in Arabidopsis, a member of the Medicago truncatula *NODULIN21* (*MtN21*) gene family), an IAA efflux transporter located in the tonoplast, can cause a dwarf phenotype^25, 27^, but its potential mechanism appears uncertain. Several studies report that intracellular auxin transporters share conserved transmembrane helices, though their amino acid sequences are quite divergent^25, 26, 28^.

For woody plants, height largely relies on secondary growth of vascular tissue in the stem. The biosynthesis of the three main components of a secondary cell wall, cellulose, hemicellulose and lignin, is the key factor affecting plant height^29, 30^. In poplar, disfunctions of the genes *CesA7A* and *CesA3D* encoding cellulose synthases, *GAUT12* encoding galacturonosyltransferase, and *LTF1* encoding a transcription factor involved in regulation of lignin biosynthesis, gives rise to dwarfism because of the disruption of normal cellulose, hemicellulose and lignin formation, respectively^31–33^. However, the molecular mechanisms behind dwarfism in woody plants remain largely unknown.

Castor bean (*Ricinus communis* L., 2n = 20) is, a typical woody oilseed crop, and it is economically important due to its seed oils being abundant in ricinoleic acids as well as being broadly used in industry for making aviation oil, lubricants, nylon, inks, adhesives and biodiesel^34–36^. Owing to the increased demand on castor oils supply, there is an immediate need to create improved varieties that can give higher yields in agriculture. Dwarfism of castor is a crucial direction to enhance yield in breeding^4, 37^. In particular, most cultivated castor varieties are 1.5–2 m in height, domesticated from the wild perennial woody tree (up to 5–7 m in height). Dwarfism is one of the main drivers of artificial selection during castor cultivation and domestication^38^. However, the physiological and molecular basis of dwarfism remains unknown in castor bean. Castor could be an ideal system to dissect the physiological and molecular mechanism underlying height and dwarf formation in woody plants.

Based on the construction of recombination inbred lines (RILs) and the segregation of given traits, the identification of key genes responsible for a given traits has been applied in diverse crops using a high throughput sequencing technology^39–41^. In particular, combining RIL population construction and the high-density SNP association analysis, bulked segregant analysis (BSA) has provided an efficient and accurate method to identify key target genes responsible for a given traits in diverse plants^42–45^. Generally, F2 progeny is sufficient for segregant bulk construction and analysis, therefore the BSA method is often considered as rapid and efficient compared to quantitative trait locus (QTL) mapping and (GWAS)^42, 46^. In this study, we compared cell size within xylem and phloem tissues and cell division within cambia tissue, and performed a comprehensive BSA analysis, detecting several genes that may be responsible for controlling plant height and therefore the dwarfing trait in castor bean. In particular, we found that the gene *Rc5NG4-1*, which we show controls IAA transport, might have been targeted by selection and is responsible in part for plant height variation in castor bean. This study provided novel insights into the physiological and molecular mechanisms of the dwarfing trait for a woody non-timber economic plant, with implications for breeding dwarf varieties in castor bean.

## Results

### Phenotypic variation of plant height in castor bean

**Figure 1:**
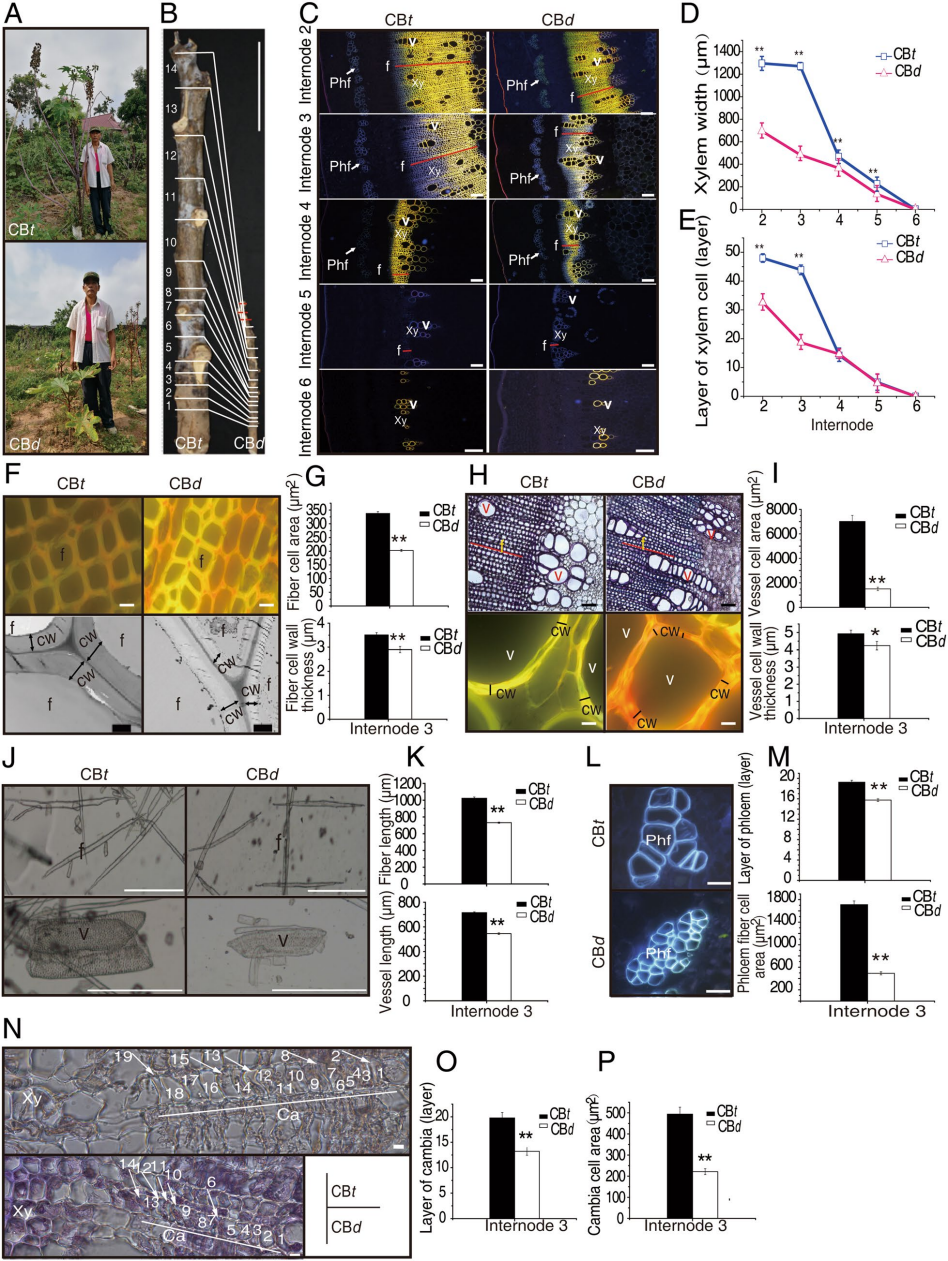
Phenotypic variation between CB*t* and CB*d*. (**A**) Plant height of CB*t* and CB*d* at maturity. (**B**) Differences in height of the primary raceme and number of nodes on main stem between CB*t* and CB*d*. Internodes numbers are marked from root end to shoot end. The red lines in CB*d* indicate extra internodes absent from CB*t*. Scale bar = 20 cm. (**C**) Fluorescence micrographs of cross section of Internodes 2–6 stained with 1% saffron and observed under ultraviolet scanning. Scale bar = 200 μm. (**D**) and (**E**) The xylem width and number of xylem layers using (**C**). (**F**) Fluorescence micrographs of fiber cell (upper panel) and transmission electron micrographs of fiber cell wall (lower panel) in cross section of Internode 3. The upper panels were obtained as in (**C**), scale bar = 10 μm. In the lower panels, black lines with double arrows indicate fiber cell wall thickness of certain fiber cells. Scale bar = 2 μm. (**G**) Fiber cell area and fiber cell wall thickness from (**F**). (**H**) Micrographs of vessel cells (upper panel) and fluorescence micrographs of vessel cell wall (lower panel) in cross section of Internode 3. In the upper panel, the graphs were observed as (**C**) but scanning under bright optic, scale bar = 100 μm. In the lower panel, black lines indicate the cell wall thickness of single vessel cell, scale bar = 10 μm. (**I**) The vessel cell area and vessel cell wall from (**H**). (**J**) Micrographs of fiber and vessel cell separated by 50% acetate acid from Internode 3. Scale Bar = 500 μm. (**K**) The length of fiber and vessel cell from (**J**). (**L**) Fluorescence micrographs of phloem fiber cells of Internode 3. The graphs scanned as (**C**), scale bar = 50 μm. (**M**) Measurement of phloem cell area and the cell layers within Internode 3 were depended on (**L**). (**N**) Cross section of cambia within Internode 3 scanned by fluorescence microscopy under bright optic. Cell layers are numbered from phloem end to xylem end. Scale bar = 10 μm. (**O**) and (**P**) Number and area of cambia cell from (**N**). In figure C-P, CB*t* and CB*d* seedlings with seven internodes were used cytological observation. Xy: xylem, v: vessels, f: fibers, Phf: phloem fibers, cw: cell wall. Results are means ± SD of at least three independent plants from each variety, and significance determined by Student’s *t-*test, * *p* ≤ 0.05, ** *p* ≤ 0.01.

The height of cultivated castor varieties usually varies from 1.5 to 2 m**.** During a broad field survey and castor bean germplasm collection, we collected a dwarf germplasm (CB*d*) with the average plant height (PH) of 45.67 ± 23.76 cm at maturity. To dissect the potential physiological and molecular mechanism of dwarfing in castor bean, we first compared CB*d* and a common castor variety (ZB159), called CB*t* here, with the average height of 236.5 ± 19.68 cm, at maturity (Fig. 1A). We measured several important traits related to height, including vertical height of the secondary branching (VHSB), height of primary raceme (HPR), number of node on main stem (NN), diameter of main stem (DMS), and average length of internodes (ALI). As shown in Fig. 1B and Table S1, the values of VHSB, HPR, DMS and ALI in CB*t* were greater than that in CB*d*, but CB*d* has more nodes on the main stem than CB*t*. ALI was substantially and significantly difference (*p* = 1.02*e*-6) between CB*t* and CB*d*, therefore the length of internodes is one of the main differences underlying height difference between two varieties.

We conducted cytological observations of cell size, cell number and cell layers within xylem, cambia and phloem tissues from developing stems of CB*t* and CB*d* within seven internodes from the bottom of the stem (Figure S1). Since it is sometime variable and difficult to delimit Internode 1 and Internode 7, we excluded these and examined Internode 2 to Internode 6. Xylem tissue was not developed at the younger internodes (Internodes 5 and 6). The more visible xylem tissues including the width and cell layers were observed in Internodes 2 to 4, with visible in both CB*t* and CB*d* (Fig. 1C).

The width and cell layers of xylem tissues at Internodes 2 and 3 exhibited substantial and significant differences between CB*t* and CB*d* (Fig. 1D,E), whereas these figures were very similar at Internodes 4–6, suggesting that developmental difference between CB*t* and CB*d* might start in Internode 3. Comparing the cell size and cell wall thickness of fibers and vessels in xylem tissues at Internode 3 revealed that cell size, cell wall thickness and longitudinal cell length of fibers and vessels were significantly different between the two accessions (Fig. 1F–K). The cell size of phloem fibers was greater and the number of cell layers of phloem more numerous in CB*t* than in CB*d* at Internode 3 (Fig. 1L,M). Similarly, the cell size and number of cell layers of cambia tissue were significant greater in CB*t* compared to CB*d* at Internode 3 (Fig. 1N–P). These observations clearly suggest that the difference in cell size across xylem, cambia and phloem as well as difference of meristematic capacity of cambia tissue might all be important factors that cause the variation in plant height.

### BSA-seq identified QTL regions associated with plant height

**Figure 2:**
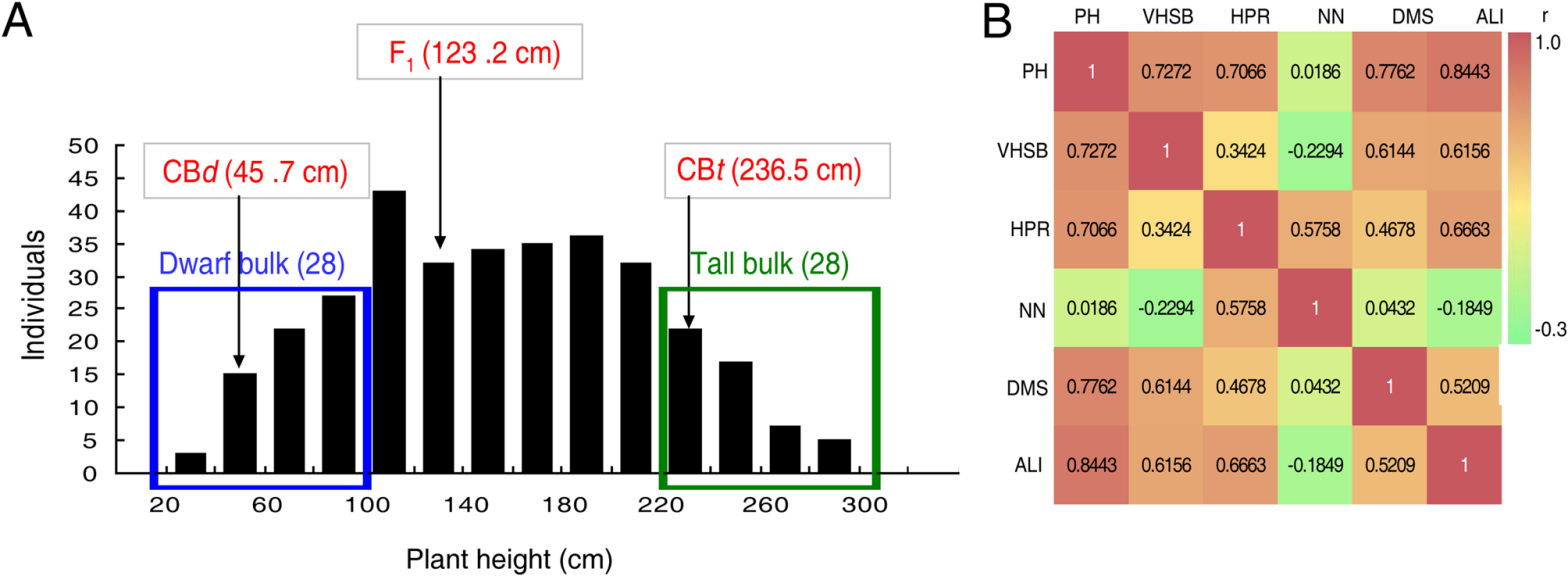
The segregation of plant height in the F_2_ population and the correlation between six height-related phenotypes. (**A**) Frequency distribution of plant height in 330 F_2_ individuals at maturity. The average values of plant height for CB*t*, CB*d* and F_1_ hybrids are shown in red text. The 28 shortest individuals (< 100 cm) were selected as the dwarf bulk and the 28 tallest individuals (> 220 cm) were selected as the tall bulk. (**B**) Correlation coefficient (*r*) among six traits in the F_2_ population. PH: plant height, VHSB: vertical height of the secondary branching, HPR: height of primary raceme, NN: number of nodes on main stem, DMS: diameter of main stem, ALI: average length of internodes on main stem.

To identify genes potentially responsible for the variation in height in castor bean, CB*d* (♀) and CB*t* (♂) were crossed to generate F_1_ hybrids. The average height of F_1_ hybrids was 123.2 cm. After selfing the F_1_, we obtained an F_2_ population comprising 330 individuals. Plant height varied from 34 to 286 cm in F_2_ population and exhibited a normal distribution (Fig. 2A), suggesting polygenic control of plant height in this population. We measured five traits in addition to PH (VHSB, HPR, NN, DMS and ALI) in the F_2_ population. ALI (*r* = 0.8443) and DMS (*r* = 0.7762) were tightly correlated with plant height, whereas NN was not correlated with plant height (r = 0.0186) (Fig. 2B). These observations were consistent with our above findings that greater PH is caused in part by the larger cell size in the xylem and phloem tissues, and the stronger meristematic capacity of cambia tissue within internodes.

**Figure 3:**
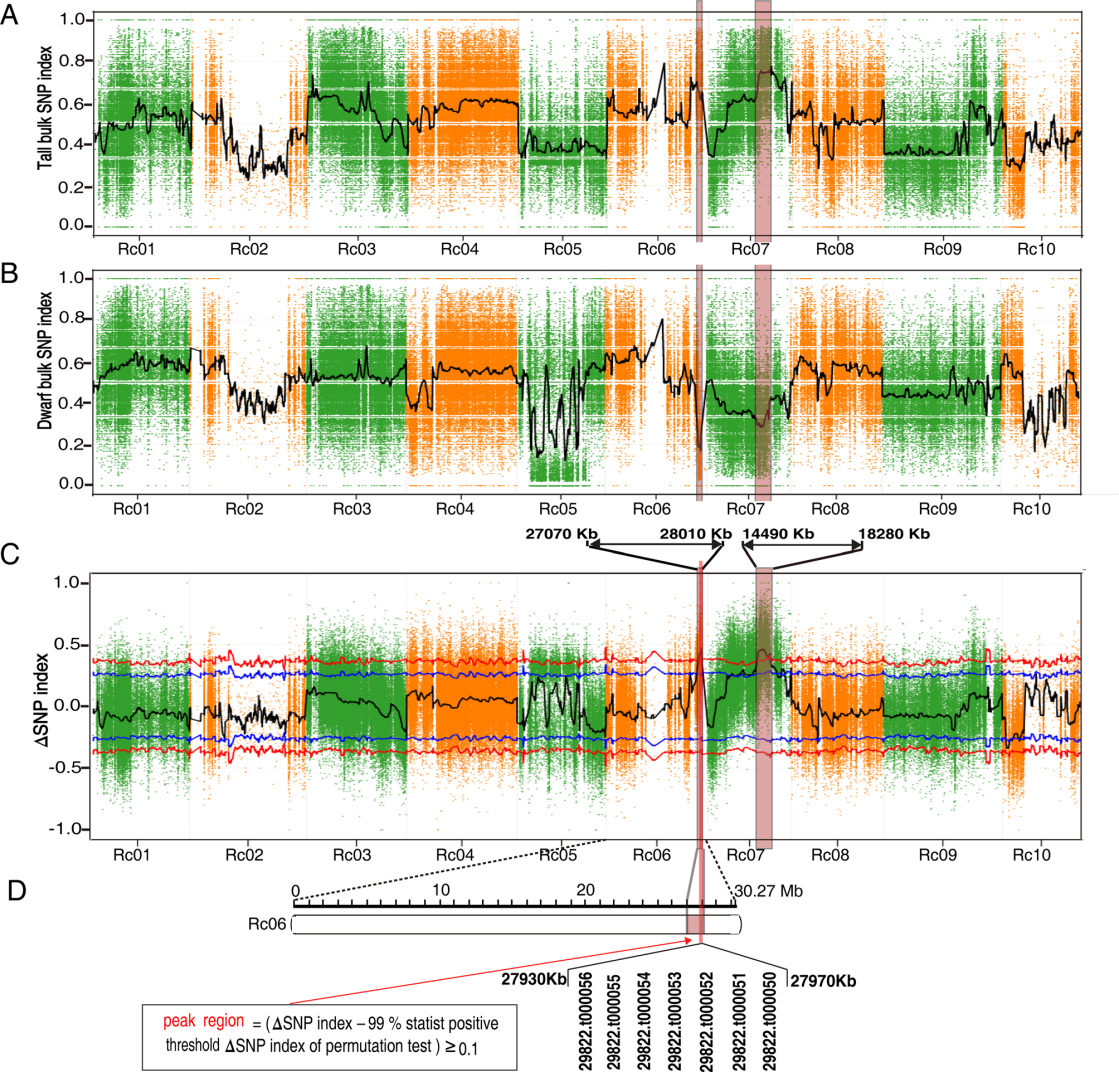
BSA for QTLs contributed to plant height using 1 Mb sliding windows with a step size of 10 Kb. SNP-index of (**A**) tall bulk and (**B**) dwarf bulk. The black curve line represents the average SNP index of each window. (**C**) ΔSNP index. The ΔSNP index of each SNP was computed as the difference between the tall SNP index and the dwarf SNP index. The black curve represents the average of ΔSNP index of each window, blue and red lines represents the 95% and 99% statistical confidence intervals, respectively, based on 1000 permutation tests to each window under the null hypothesis of no QTL. The pink rectangles in (**A**), (**B**) and (**C**) represent the QTLs (*p* value < 0.01). The red vertical line in (**C**) is the QTL1 peak region. Rc01-Rc10 in abscissa are the 10 castor bean chromosomes according to^41^. (**D**) The peak candidate region located under QTL1 covers seven genes. The pink rectangle and red vertical line are the same as in (**C**).

Based on the segregation of phenotype, we selected the 28 shortest individuals (average height 71.32 ± 20.14 cm) as the dwarf bulk and the 28 tallest individuals (average height 254.54 ± 21.44 cm) as the tall bulk (Fig. 2A) for genome sequencing to identify loci potentially associated with plant height. The sequencing generated 83,983,462 and 79,741,716 high quality clean reads for the dwarf bulk and tall bulk, respectively, a depth exceeding 32-fold of the genome (Table S2). After aligning the clean reads to the castor reference genome (http://castorbean.jcvi.org) and variant calling using GATK 3.4, 1,027,623 genomic SNPs were identified from the parents and two bulks. The physical positions of SNP were aligned and annotated by ANNOVAR according to our previously constructed castor chromosomes^41^. In total, 300,225 SNPs homozygous within but different between the parents were identified, and their ΔSNP index and the average ΔSNP index within each 1 Mb window (with 10 kb step size) were calculated. Based on the 99% statistical confidence intervals (permutation tests under the null hypothesis of no QTLs), two regions, QTL1 (0.94 Mb in length; 27.07–28.01 Mb on chromosome Rc06) and QTL2 (3.79 Mb in length; 14.49–18.28 Mb on chromosome Rc07), were identified as genomic regions associated with height in castor bean (Fig. 3A–C). We chose to focus on the region on Rc06 (27.93–27.97 Mb) where the difference was greatest between the ΔSNP index (0.463) and the 99% statistical threshold (0.350) based on the recommendation of reference^42^ (Fig. 3D).

### Candidate genes associated with plant height were identified

In total, 487 genes were identified in the *QTL1* and *QTL2* regions (Table 1). After filtering out the 78 genes without ΔSNP index (≥ 0.5) and the 84 genes with SNPs in intergenic regions, we obtained 325 genes with SNPs located within the gene or within 2000 bp of the coding region (Figure S2A–B). The 325 genes were considered candidate genes associated with castor bean height. Gene ontology (GO) analysis showed 146 of 325 genes were functionally involved in cell component (Figure S2C). Based on the *p* value of GO enrichment analysis, we found that these candidate genes were mainly enriched in lignin metabolism, secondary metabolism and phenylalanine metabolism (Figure S2D). KEGG enrichment analysis found that the majority of candidate genes were enriched in hormone signal transduction and secondary metabolism processes (Figure S2E).

**Table 1:** Details of two QTL associated with plant height.

	Position in chromosomes	Interval/bp	Region/Mb	Scaffold/No	SNP/No	Gene/No
*QTL1*	Rc06	27,070,001–8,010,001	0.94	1	1501	140
*QTL2*	Rc07	14,490,001–8,280,001	3.79	29	5006	347

We identified where in the gene the SNPs were located and found non-synonymous polymorphisms in 118 genes that differentiated CB*d* and CB*t* (Figure S2B). To reduce the potentially spurious associations between SNPs and height as far as possible we applied a strict criterion with a ΔSNP index ≥ 0.6, as suggested by Takagi et al.^42^, to sort out target candidate genes. Based on properties of the amino acids polymorphisms, 29 target candidate genes with non-synonymous SNPs (ΔSNP index ≥ 0.6) were selected for further investigation, including 6 and 23 genes located at *QTL1* and *QTL2*, respectively (Table 2).

**Table 2:** The candidate genes potentially controlling plant height in castor bean identified in QTL.

Gene ID	QTL region	ΔSNP index ≥ 0.6	Functional annotation
29,822.t000132	*QTL1*	0.668	Conserved hypothetical protein
29,822.t000126	*QTL1*	0.682	HIPL1 protein precursor, putative
29,822.t000107	*QTL1*	0.653	Nop14, putative
29,822.t000095	*QTL1*	0.62	Mannosidase alpha class 2a, putative
29,822.t000076	*QTL1*	0.707	Peroxidase 31 precursor, putative
29,822.t000050	*QTL1*	0.628	Auxin-induced protein 5NG4, putative
29,767.t000004	*QTL2*	0.707;0.607	NADPH fad oxidoreductase, putative
29,767.t000005	*QTL2*	0.616	Pentatricopeptide repeat-containing protein, putative
29,846.t000009	*QTL2*	0.617	Pectin acetylesterase, putative
29,846.t000008	*QTL2*	0.674	Protein phosphatase 2c, putative
29,846.t000007	*QTL2*	0.727	Conserved hypothetical protein
29,846.t000002	*QTL2*	0.695	Ubiquitin ligase E3 alpha, putative
27,798.t000017	*QTL2*	0.643	Conserved hypothetical protein
27,798.t000018	*QTL2*	0.808	Conserved hypothetical protein
27,798.t000020	*QTL2*	0.671	Conserved hypothetical protein
29,973.t000023	*QTL2*	0.681	ATP binding protein, putative
29,973.t000005	*QTL2*	0.629	Cytoplasmic dynein light chain, putative
29,969.t000005	*QTL2*	0.654	Conserved hypothetical protein
28,308.t000003	*QTL2*	0.658	Transcription factor, putative
29,568.t000010	*QTL2*	0.630	Amino acid transporter, putative
29,568.t000002	*QTL2*	0.684;0.610	Cyclin d, putative
29,701.t000006	*QTL2*	0.700	Symplekin, putative
29,701.t000011	*QTL2*	0.611	Translation initiation factor if-2, putative
29,701.t000013	*QTL2*	0.637	Isoamyl acetate-hydrolyzing esterase, putative
29,701.t000019	*QTL2*	0.667	Conserved hypothetical protein
28,345.t000007	*QTL2*	0.740	Tropinone reductase, putative
28,345.t000005	*QTL2*	0.631	Ribulose bisphosphate carboxylase/oxygenase activase 1
28,345.t000004	*QTL2*	0.634	Transferase, transferring glycosyl groups, putative
28,345.t000003	*QTL2*	0.697	Hypothetial protein

### *Rc5NG4-1* was demonstrated as a candidate gene controlling castor height

**Figure 4:**
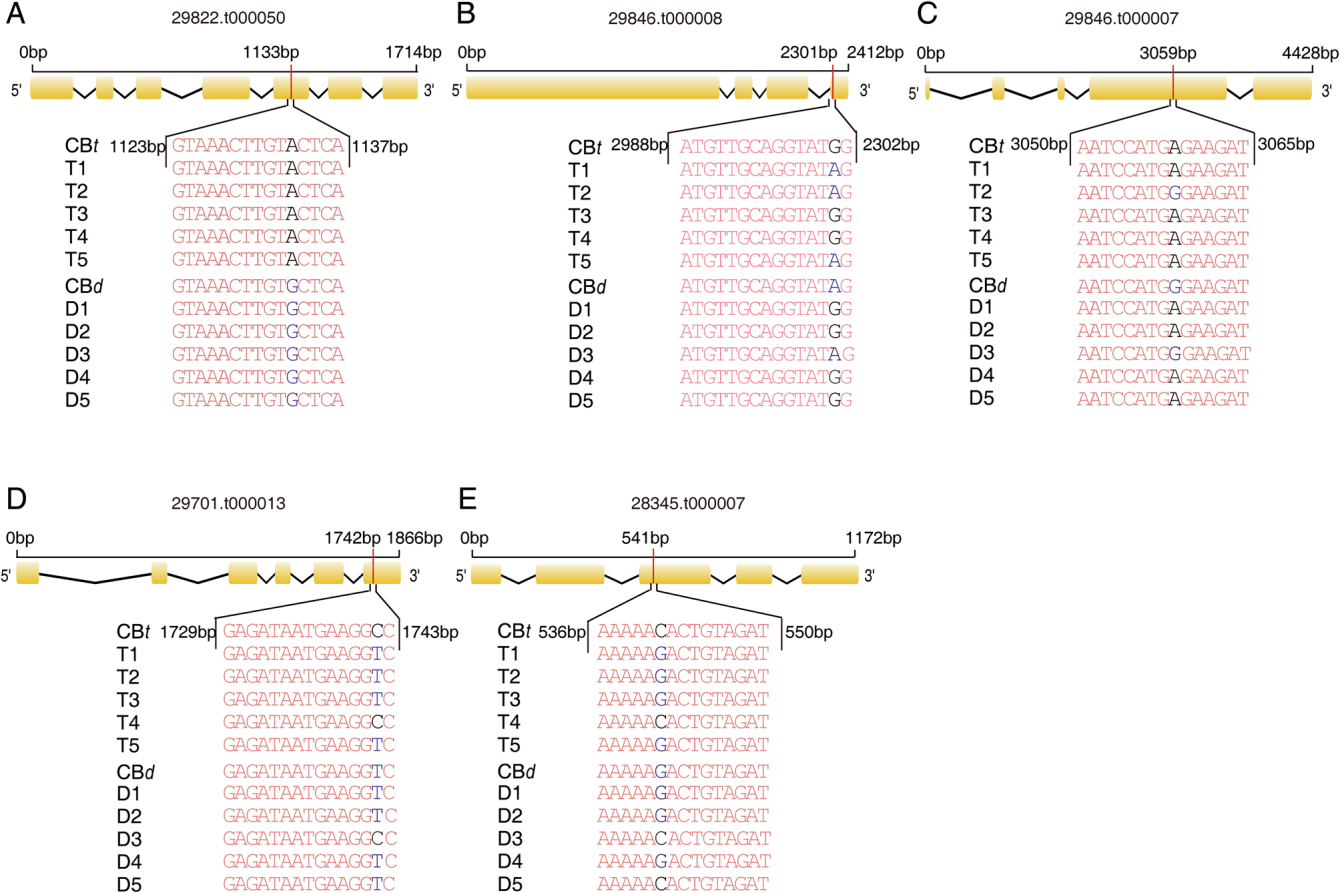
ΔSNP index ≥ 0.6 non-synonymous SNPs identification for five candidate genes obtained from 10 castor varieties. (**A**)–(**E**) The exon (box) and intron (line) structure of 29,822.t000050, 29,846.t000008, 29,846.t000007, 29,701.t000013 and 29,345.t000007 and the genotypes of non-synonymous SNPs with ΔSNP index ≥ 0.6 in tall (T) and dwarf (D) accessions determined using PCR and sequencing. Collection location and plant height of the 10 varieties are shown in Table S4.

To investigate whether the SNP variants were associated with plant height among different germplasms, we determined the genotypes of five wild castor bean accessions with average height of 539.2 ± 31.76 cm, and five cultivated castor bean accessions with average height of 153.4 ± 13.16 cm (Table S3). Among the 29 candidate genes, based on their putative functional annotation directly or indirectly related to cell wall formation (tightly associated with dwarf phenotype), five (29,822.t000050, 29,846.t000008, 29,846.t000007, 29,701.t000013 and 28,345.t000007) were selected to further investigate the genotypes of non-synonymous SNP with ΔSNP index ≥ 0.6 in this extended germplasm (Table 2). As shown in Fig. 4, we found only the non-synonymous A/G SNP at position 1133 bp in gene 29822.t000050 to be fixed between the tall and dwarf germplasm. These results strongly suggest that gene 29822.t000050 might be an important candidate associated with height. However, we noted that the ΔSNP index of 29,822.t000050 was not the highest among identified candidate genes based on the statistic analysis (see Table 2).

**Figure 5:**
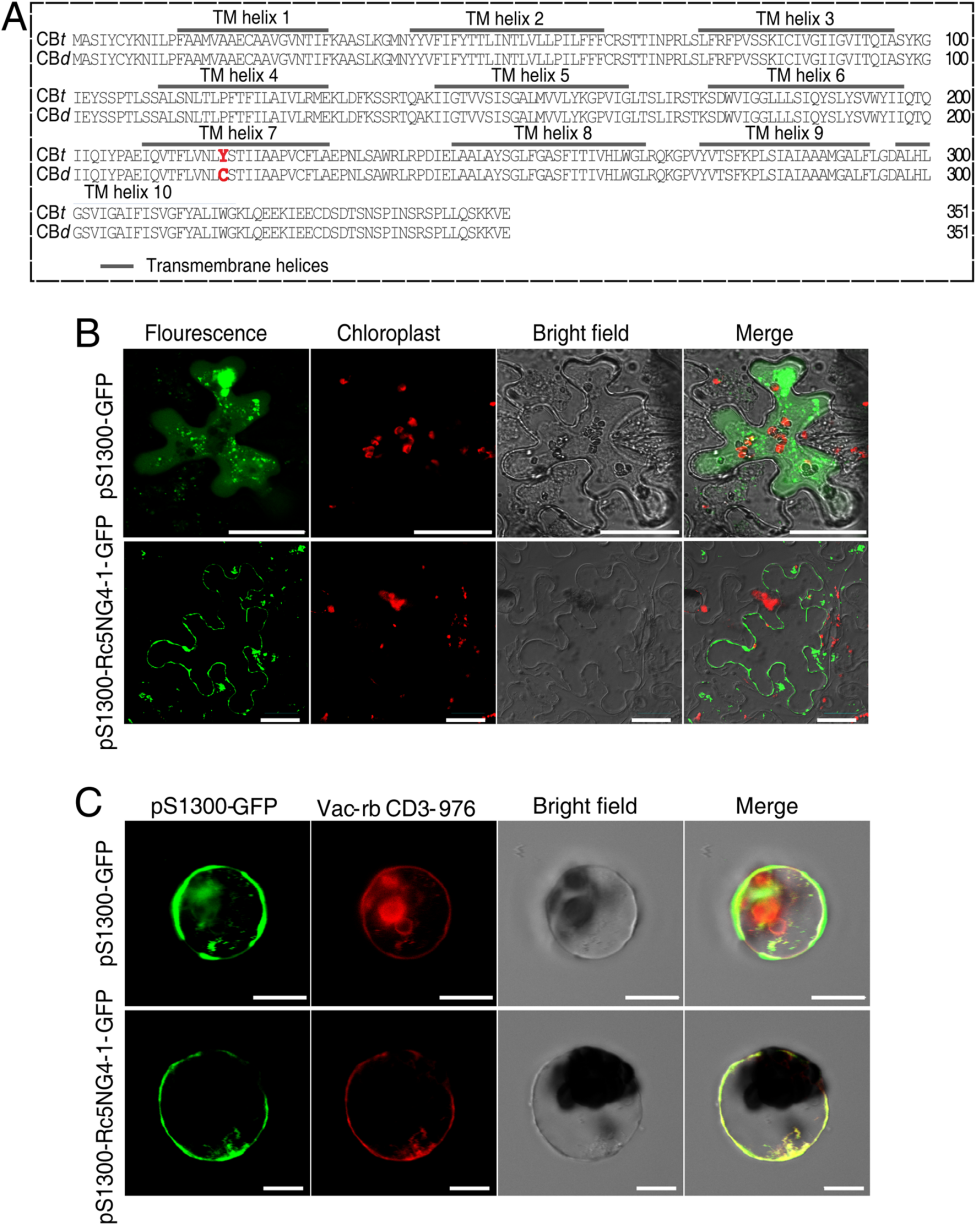
Protein structure and subcellular localization of Rc5NG4-1. (**A**) Protein sequence structures and transmembrane (TM) domains of Rc5NG4-1 in CB*t* and CB*d* predicted using TMHMM. The 218th amino acid change are marked red, which induced by the non-synonymous SNP located in 1133 bp position of *Rc5NG4-1*. (**B**) *Rc5NG4-1*-fused GFP was expressed in hypoepidermal cell of tobacco leaves. Scale bar = 30 μm. (**C**) *Rc5NG4-1*-fused GFP was transiently co-expressed with the vacuolar membrane marker in protoplasts. Empty vector pS1300-GFP was expressed in protoplasts as a control (top panels). Vac-rb CD3-976, the vector carries tonoplast marker γ-TIP coding sequence (aquaporin with tonoplast localization). Images in bottom panels show colocalization of Rc5NG4-1 and γ-TIP. The fluorescence graphs were detected by confocal microscope in (**B**) and (**C**). Scale bar = 10 μm.

According to the annotation of 29,822.t000050, it is predicted to be an auxin induced protein 5NG4, a member of the MtN21 family^47^. As the first *5NG4*-like gene to be reported in castor bean, we named 29,822.t000050 as *Rc5NG4-1*. The non-synonymous SNP resulted in the substitution of the amino acid 218th from tyrosine (Y) in CB*t* to cysteine (C) in CB*d* (Fig. 5A). Previous studies have found that the protein of 5NG4 members have 10 transmembrane (TM) helices that often form several transmembrane secondary loop structures for facilitating IAA transportation. The Arabidopsis orthologue *AtWAT1* (*At1g75500*) functions as an auxin efflux transporter localized on tonoplast, facilitating IAA transportation from vacuole to cytosol^25^. We predicted the protein secondary structure of Rc5NG4-1 using TMHMM Server v. 2.0, found it had 10 TM domains and the amino acid substitution described above was located in TM helix 7, though did not result in a change to the TM helix number or number of hydrophilic loops (Fig. 5A). Inspection of the subcellular localization of Rc5NG4-1 in tobacco epidermal cells and Arabidopsis protoplasts, revealed Rc5NG4-1 was not localized in the plasma membrane (Fig. 5B) but was instead co-localized with the tonoplast marker (Fig. 5C). Thus, we reasonably infer that the function of *Rc5NG4-1* is involved in IAA transportation from vacuole to cytosol in castor bean. Further, while inspecting the expressional profiles of *Rc5NG4-1* among different tissues we found *Rc5NG4-1* have relatively higher expressions in root and stem, compared to that in leaf and seed (Figure S3).

### Functional confirmation of *Rc5NG4-1* involved in IAA uptake

**Figure 6:**
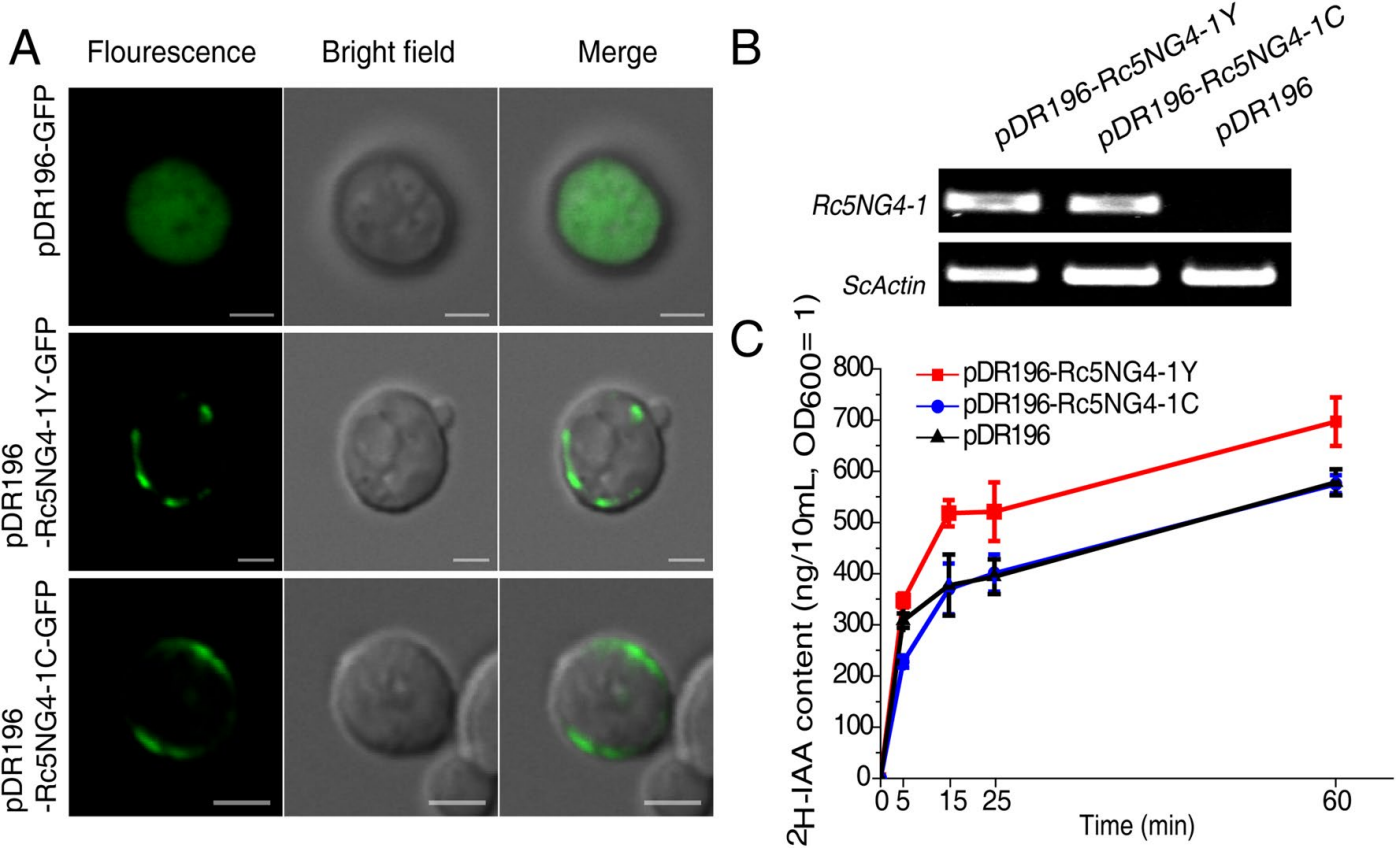
Examination of IAA uptake based on heterologously transformed yeast. (**A**) *Rc5NG4-1Y-* or *Rc5NG4-1C*-fused GFP were expressed in yeast cell and fluorescence observed by confocal microscopy. Scale bar = 2 μm. (**B**) *Rc5NG4-1Y* and *Rc5NG4-1C* expression in *Rc5NG4-1Y* transformed yeast, *Rc5NG4-1C* transformed yeast and empty vector pDR196 transformed yeast detected by RT-PCR. The upper group and lower group of blots were cropped from different part of same gels, and full-length blots are presented in Figure S4. (**C**) ^2^H-IAA uptake of the different transformed yeast strains. The values are means ± SD of three replications (transformations carried out once for each and then grow in triplicate).

To inspect whether the non-synonymous SNP that resulted in the substitution of amino acid 218 from tyrosine (Y) in CB*t* to cysteine (C) in CB*d* gave rise to functional difference in auxin transport, we performed a heterologous expression of *Rc5NG4-1* in yeast (*Saccharomyces cerevisiae*). First, we checked the subcellular localization of Rc5NG4-1Y and Rc5NG4-1C in transformed yeast strains using pDR196-Rc5NG4-1Y-GFP or pDR196-Rc5NG4-1C-GFP vectors. As shown in Fig. 6A, Rc5NG4-1Y and Rc5NG4-1C were localized in the membrane. Second, *Rc5NG4-1Y* and *Rc5NG4-1C* were heterologously expressed in yeast cells and expression of *Rc5NG4-1Y* and *Rc5NG4-1C* was confirmed using RT-PCR (Fig. 6B). The uptake capacity of free IAA (as ^2^H-IAA in the growth medium) in transformed yeast strains was assayed. The uptake rates of ^2^H-IAA were higher in yeast transformed with *Rc5NG4-1Y* than yeast transformed with *Rc5NG4-1C* from 5 to 60 min of ^2^H-IAA supply (Fig. 6C). The uptake rates of ^2^H-IAA in transformed *Rc5NG4-1C* yeast strains was almost identical to the control, suggesting *Rc5NG4-1C* may have lost the capability to uptake free IAA. These results clearly show that *Rc5NG4-1Y* and *Rc5NG4-1C* lead to functional protein divergence resulting in a difference in IAA uptake.

### Content of free IAA in CB*d* and CB*t*

**Figure 7:**
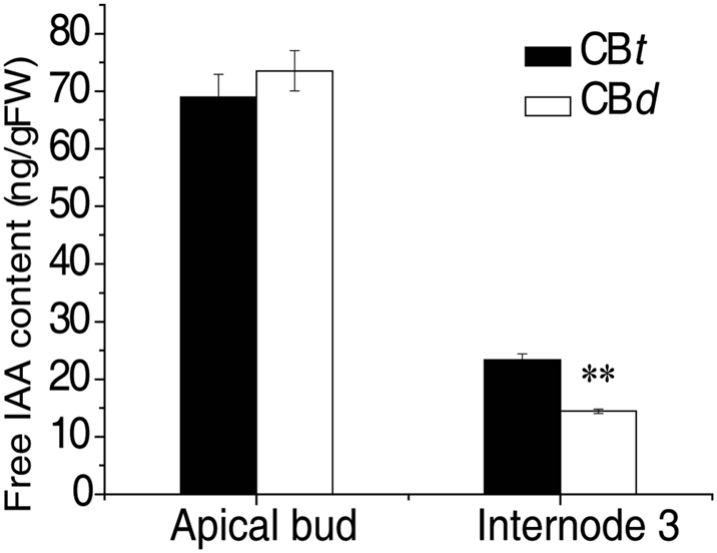
Measurement of free IAA concentration in CB*t* and CB*d*. The free IAA concentration within apical bud and Internode 3 were measured using CB*t* and CB*d* seedlings with seven internodes. Values are means ± SD of three replicate determinations, **p value ≤ 0.01(Student’s t-test).

Content of free IAA in plant tissues is usually determined by intracellular IAA transport between organelle and cytosol, cellular influx and efflux transport, and long distance transport among tissues^23, 25, 48^. To test whether the differences between the tall and dwarf phenotypes related to free IAA, we measured the content of free IAA in apical buds and Internode 3 tissues in CB*t* and CB*d*. As shown in Fig. 7, the content of free IAA did not differ between CB*d* and CB*t* in the apical bud, whereas there was significantly lower free IAA in Internode 3 of CB*d* than in CB*t* (*p* value = 0.0013, *t*-test). This result suggests that the biosynthesis of free IAA in apical bud might be similar between the two accessions, but the transport capability of free IAA from the apical bud to node is weaker in CB*d*.

## Discussion

As a key component of tree architecture, plant height is a critical agronomic trait in non-timber trees such as fruit trees and woody oilseed crops. Dwarfism is beneficial for reducing canopy size, allowing increased planting density and greater yields, lowering costs and increasing the orchard’s lifespan of non-timber trees. Dwarfism in plants is a complex trait and relatively little is known about the physiological and genetic factors that regulate dwarfing in non-timber trees. As mentioned above, castor bean is an ideal system to dissect the potentially physiological and molecular mechanisms underlying dwarfism in woody plants because of its domestication from a tall wild perennial woody tree to the shorter annual oilseed crop^38^.

Based on our phenotypic observations, dwarfism is clearly caused by shorter internodes and thinner stems, implying that stem growth of CB*d* might be developmentally repressed. Cytological observations revealed that cell growth in xylem and phloem tissues and the cell division of cambia tissue were repressed in CB*d*, resulting in smaller cells within the xylem and phloem tissues and fewer cell layer in cambia tissue compared to CB*t*. Similarly, in poplar, larger phloem and vessel tissues lead to greater plant height^33, 49^. Since cell division in cambia tissue is an important driver of radial and longitudinal growth in woody plants^50^, we infer that the capability of cell division within cambia tissue is probably a critical factor affecting the radial and longitudinal growth in castor bean internodes.

BSA is an efficient and rapid method to detect plausible candidate genes responsible for a given trait in plants from the F_2_ generation. Based on the segregation of plant height in the F_2_ generation, we detected two QTLs associated with castor bean height. Within the two identified QTLs, 325 candidate genes associated with the plant height were mainly enriched in cell component, functionally involved in hormone signal transduction and secondary metabolism processes, consistent with the requirement of plant growth and development in woody plants. Based on the non-synonymous polymorphisms and a strict criterion (ΔSNP index ≥ 0.6) we sorted out 29 candidate genes that were likely participated in regulating the formation of plant height in castor bean. Within these identified candidate genes, we sorted out a candidate gene *Rc5NG4-1* that was functionally involved in the regulation of cellular auxin transport. However, according to current analyses it is difficult to determine or rule out which candidate genes are the causal genes controlling the formation of plant height in castor bean.

Many studies have revealed that the GA signaling network is a critical factor that regulates plant growth, in some cases underlying dwarf phenotypes^2, 51, 52^. Indeed, the physiological and molecular basis of plant growth regulation and dwarfing are complex and varied across plant species. Our current study demonstrated that *Rc5NG4-1* is located in the tonoplast, and is an auxin transporter, likely capable of transporting IAA between vacuole and cytosol. Based on expressing *Rc5NG4-1* heterologously in yeast, it is clear that the uptake capacity of free IAA between *Rc5NG4-1Y* (tall allele) and *Rc5NG4-1C* (dwarf allele) is significantly different validating the different uptake and transport capacities of *Rc5NG4-1Y* and *Rc5NG4-1C*. It is likely that *Rc5NG4-1* functions in a similar way to its putative orthologue *AtWAT1* encoding an IAA transport protein in *Arabidopsis thaliana*^25^ by transferring IAA between the vacuole and cytosol to maintain the free IAA content in cytosol. It appears that the *Rc5NG4-1C* allele, whilst being expressed, was non-functional. Using *Rc5NG4-1* as a query in a BLAST search against the castor bean genome reference we found several putative paralogues (data not shown), suggesting that other copies of *Rc5NG4-*like genes might compensate for this loss-of-function, thus partially maintaining the free IAA content in cytosol. Due to the free IAA content in the apical bud being similar between CB*t* and CB*d*, and the difference in the internode tissues, we infer that long-distance free IAA transport capability from the apical bud to the internode might vary between two varieties. Since auxin affects cell division in cambia tissue and cell growth in xylem and phloem tissues^53, 54^, we reasonably infer that our observed reduction in IAA in the internode tissues in CB*d* might be an important physiological difference resulting in the dwarfing of castor bean. Based on the sequence similarity we found the orthologue *AtWAT1* of *Rc5NG4* in Arabidopsis. The function of *AtWAT1* has been verified to control plant height by affecting cellular auxin transport in Arabidopsis^25, 27^. Our results indicated that *Rc5NG4* is likely a causal gene that gives rise in phenotypic variation of plant height in woody castor bean. However, such study is very limited in other plants. Whether or how the mutant *Rc5NG4-1C* directly influences cellular influx and efflux of IAA and the long distance transportation of free IAA remain unknown.

## Conclusions

In this study, we first found divergent cell growth in xylem and phloem tissues and divergent cell division in cambia tissues, suggesting the potential physiological basis of dwarfing in castor bean. Two QTLs associated with plant height which cover 325 candidate genes were identified using bulked segregant analysis sequencing from an F2 population derived from a tall castor variety CB*t* and a dwarf castor variety CB*d*. In particular, we detected a gene *Rc5NG4-1* (encoding an IAA transporter protein localized in tonoplast) as a responsible candidate for controlling dwarfism formation in woody oilseed crop castor bean. This study provided novel insights into the physiological and molecular mechanisms of dwarfing in woody non-timber economic plants, facilitating the genetic improvement to create dwarf varieties in castor bean.

## Materials and methods

### Plant materials and phenotype survey

The castor bean varieties CB*d* (dwarf) and CB*t* (tall) were developed by self-pollination controlled for at least three generations, provided by Zibo Academy of Agriculture Sciences, Shandong Province. The varieties CB*t* and CB*d* were genetically independent, and were applied as parents for creating the F_2_ population in 2013–2016. The F_1_ hybrids were produced by crossing between CB*t* (♂) and CB*d* (♀) in 2016, and F_2_ recombinant population that derived from selfing the F_1_ hybrids. Parents and two populations of offspring were grown in the experiment field managed by Xishuangbanna Tropical Botanical Garden, Chinese Academy of Sciences in Mengla County, Xishuangbanna, Yunnan province (101° 25′ E, 21 41′ N) in 2017, each planted 1 m apart. Six traits, including plant height (PH), vertical height of the secondary branching (VHSB), height of primary raceme (HPR), number of node on main stem (NN), diameter of main stem (DMS), and average length of internodes on main stem (ALI), were measured after seed ripening for parent lines and each individual of the F_2_ population. Only PH was measured for F_1_ hybrids. The DMS was the diameter of the middle internode of main stem. ALI was defined as the ratio of HPR to NN in main stem.

### The observations of cytology

The CB*t* and CB*d* seedlings were grown in a plastic basin (the ratio of nutrient soil, soil and perlite was 3:1:1, v/v) in the greenhouse (30 °C, light/dark was 12 h/12 h) and individuals at the same maturity stage (seven internodes) were used in the anatomical survey, transmission electron microscopy and maceration of fibers and vessels within stem.

The stem from CB*t* and CB*d* seedlings were split into 1 cm sections manually using a razor blade, immersed in 30% glycerin, infiltrated in a vacuum environment (0.08 MPa) for 30 min, and sustained in 30% glycerin. The 1 cm samples were then cut into blocks (2 mm × 2 mm) and fixed into the chuck with refrigerant. Then, sections of 15–25 μm thickness were sliced continuously using freezing microtome (Leica, CM3050S, Germany), transferred onto microslides and rinsed until the refrigerant was removed completely. After this, the sections were stained with 1% saffron (1 g saffron dissolved in 100 ml 50% ethanol, m/v) for one minute, and the residual dye was removed using 50% ethanol. After rehydration with sterile water, the sections were inspected with a fluorescence microscope (Leica Microsystems, DM5508, Germany) under white light or ultraviolet light to scan the xylem tissue and the phloem fibers^55^. Images were captured using a Leica camera (Leica Microsystems, DFC450C, Germany), and cell area, cell layer and cell wall thickness were measured with Image J software (version 1.46). All cells from each image were measured, and the values from at least five images of at least three plants were measured.

Transmission electron microscopy was used for inspecting cell thickness of xylem in Internode 3. The xylem tissue was shaped to 1 mm × 1 mm blocks, fixed in 2.5% glutaric dialdehyde for at least 24 h and immersed in 1% osmium acid for 12 h under 4 °C. After dehydration in an acetone series (final concentration of 90% acetone) and embedment in resin spur, the 40 nm-thick sections were sliced by microtome (Leica-R, Germany). The sections were dried at room temperature and stained using uranylacetate and lead citrate. The stained sections were inspected under an electron microscope (JEOL Ltd, Tokyo, Japan), and images captured by an Olympus-SIS Megaview camera (Olympus Soft Imaging Solutions GmbH, Münster, Germany). Measurement of the cell wall followed the above method.

The xylem in the Internode 3 of seedlings was macerated to separate fibers and vessels for measuring length. The maceration method was similar to that of Biswal et al.^33^. The tissue was macerated for 10 h at 90 °C with buffer (50% acetate acid and 3% hydrogen peroxide) in 2 mL polypropylene tubes. After washing with sterile water, the fibers and vessels were observed by microscope (Leica Microsystems, DM5508, Germany). Image capturing and trait measurements were followed above protocols.

### DNA extraction and BSA analysis

DNA were isolated from young leaves of single plants in parents, the 28 tallest plants and the 28 most dwarf plants using a plant genomic DNA extraction Kit (TIANGEN, Beijing, China). The tall bulk and dwarf bulk were constructed by mixing equivalent DNA amounts from each of 28 tall plants and each of 28 dwarf plants, respectively. Pair-end sequencing libraries were generated for the two parents, the tall bulk and the dwarf bulk and sequenced using Illumina HiSeq™ 2500 (Illumina Inc, San Diego, CA, United States). High quality clean 150 bp reads (HQ clean reads) were obtained by filtering out reads with adapters, with > 10% unidentified nucleotides (N) and with quality scores (Q) ≤ 20 bases accounting for more than 50%. The depth for two parents and two bulks were above 32-fold (Table S2).

HQ clean reads were aligned against the reference genome (http://castorbean.jcvi.org) using the Burrows–Wheeler Aligner (BWA) software (settings: mem 4 -k 32 -M). After duplicates marking by picard (1.129), variants (SNPs) were identified using UnifiedGenotyper of GATK (3.4–46) and filtered using VariantFiltration (settings: -Window 4, -filter “QD < 4.0 || FS > 60.0 || MQ < 40.0”, -G filter “GQ < 20”) of GATK. The physical positions of variants were aligned and annotated by ANNOVAR according to the 10 chromosomes constructed by Yu et al.^41^. The SNP index of the tall bulk and dwarf bulk were calculated using homozygous and biallelic SNPs that differentiate the two parental genomes and were found at depth > 2. ΔSNP index were calculated by subtracting SNP index of dwarf bulk from SNP index of tall bulk, after filtering out SNPs with read depth < 6 and SNP index < 3 in the two bulks. The average SNP index and ΔSNP index were computed using 1 Mb sliding window with 10 kb step size, and the 95% and 99% statistical confidence intervals of ΔSNP index were obtained from 1000 permutation tests of each window under the null hypothesis of no QTLs. Windows with < 10 SNPs were ignored. Nr description, GO term and KEGG pathway were performed to candidate genes.

### Association analysis of SNP genotype and plant height phenotype

Five tall (wild varieties) and five dwarf (cultivars) castor bean accessions collected from different areas were examined for their SNP genotypes (see Table S3). They were planted in an experiment field at Kunming Institute of Botany, Chinese Academy of Sciences in Kunming, Yunnan Province (102° 10′ E, 26 22′ N). We surveyed the plant height of each variety after maturity and collected young leaves of each and performed genomic DNA extraction as above. To obtained the genotype information of non-synonymous SNP with ΔSNP index ≥ 0.6 located at five target genes in each variety, normal PCR was carried out for each genomic DNA sample using specific primers (listed in Table S4) binding with the flanking sequence of each SNP, with reaction protocol 95 °C for 2 min, 30 cycles of 95 °C for 30 s, 55 °C for 15 s, and 72 °C for 30 s. Amplicons were purified using a gel extraction kit (OMEGA BIO-TEK, Norcross, USA). The genotypes of SNP were captured via Sanger sequencing using the same primers as the PCR amplification.

### Construction of subcellular localization and over-expression vectors

The cDNA (1062 bp, sequence see Supplementary file 1) of *Rc5NG4-1Y* and *Rc5NG4-1C* were amplified from Internode 3 of CB*t* or CB*d* seedlings, respectively, at the seven internode stage, using primers 5NG4-1-cDNA-F (5′-ATGGCTTCAATATACTGTTACAAG-3′) and 5NG4-1-cDNA-R (5′-TTATGCATCTTCAACTTTTTTGCTT-3′). The cDNA sequences from different castor varieties were recovered from 1% (m/v) agarose gels and purified with a Gel Extraction kit (OMEGA BIO-TEK, Norcross, USA). The cDNA sequences of *Rc5NG4-1* from CB*t* and CB*d* were cloned into pEASY-T1 Cloning vector (Transgen, Beijing, China) to construct pEASY-T1-5NG4-1Y and pEASY-T1-5NG4-1C.

pSuper1300-GFP (pS1300-GFP) used in subcellular localization research in tobacco and protoplast of *Arabidopsis thaliana* was a kind gift of Professor Pengtao Wang (State Key Laboratory of Cotton Biology, Henan Key Laboratory of Plant Stress Biology, College of Life Sciences, Henan University). When the pS1300-5NG4-1-GFP was constructed, the coding sequence of *Rc5NG4-1* in CB*t* was amplified from pEASY-T1-5NG4-1Y with primers 5′-CTGCAGGGGCCCGGGGTCGACATGGCTTCAATATACTGTTACAAG-3′ and 5′-CATGGTACCGGATCCACTAGTTGCATCTTCAACTTTTTTGCTT-3′ and cloned into pS1300-GFP vector cut with *Sal*Ι and *Spe*Ι. The cDNA of Rc5NG4-1Y-GFP and Rc5NG4-1C-GFP (GFP fused in carboxy terminal) were amplified from pS1300-5NG4-1-GFP and pS1300-5NG4-1C-GFP (the methods followed pS1300-5NG4-1-GFP construction, but amplified 5NG4-1 from pEASY-T1-5NG4-1C) with primers 5′-AGTGGATCCCCCGGGCTGCAGATGGCTTCAATATACTGTTACAAG-3′ and 5′-GGGCCCCCCCTCGAGGTCGACTTACTTGTACAGCTCGTCCATG-3′, then were cloned into the pDR196 yeast over-expression vector (Transgen, Beijing, China) and then cut with *Pst*Ι and *Sal*Ι, respectively, to construct the vectors of pDR196-5NG4-1Y-GFP and pDR196-5NG4-1C-GFP. When pDR196-5NG4-1Y and pDR196-5NG4-1C were constructed, the coding sequences of Rc5NG4-1Y and Rc5NG4-1C were amplified from pEASY-T1-5NG4-1Y and pEASY-T1-5NG4-1C, respectively, with primers 5′-AGTGGATCCCCCGGGCGCAGATGGCTTCAATATACTGTTACAAG-3′ and 5′-GGGCCCCCCCTCGAGGTCGACTTATGCATCTTCAACTTTTTTGCTT-3′, and were cloned into pDR196 cut with *Pst*Ι and *Sal*Ι.

### The transformation of tobacco leaves, Arabidopsis protoplast and yeast

The pS1300-5NG4-1-GFP vector was transformed into *Agrobacterium* (GV3101). The transient expression in *Nicotiana benthamiana* by the *Agrobacterium*-mediation followed Chatre et al.^56^. pS1300-5NG4-1-GFP vectors (1 μg/μl) were transiently expressed in Arabidopsis protoplast with the Vac-rb CD3-976 vector^57^, following Yoo et al.’s method of transient gene expression in mesophyll protoplasts^58^. The preparation of yeast competent cells (INVSc1 strain), and yeast transformation of pDR196 vectors were carried out using Frozen-ER Yeast Transformation ΙΙ kit (ZYMO RESEARCH, Beijing, China) following the manufacturers protocol. The fluorescence of transformed tobacco leaves, protoplasts and yeast were examined using a confocal microscope (Fluoview fv1000, OLYMPUS).

### RNA extraction and RT-PCR in yeast cell

RNA extraction from yeast cells followed the manufacturer’s protocol of Yeast RNA kit (OMEGA BIO-TEK, Norcross, USA) using 4 mL yeast cells, OD_600_ = 1.0. Genomic DNA removal and cDNA synthesis used the One-Step gDNA Removal and cDNA synthesis SuperMix (Transgen, Beijing, China). The semi-quantitative reverse transcription PCR (RT-PCR) was used to detect the expressional level of *5NG4-1Y* and *5NG4-1C* in yeast cell. The protocol of RT-PCR was 95 °C for 2 min, 30 cycles of 95 °C for 30 s, 60 °C for 15 s, and 72 °C for 10 s. *ScACTIN* was treated as the reference gene^25^. The primer sequences of RT-PCR are listed in Table S2.

### IAA testing after yeast uptake and IAA concentration determination in castor bean

Before the yeast uptake experiments, the *Rc5NG4-1Y* over-expression strain, *Rc5NG4-1C* over-expression strain and pDR196 transformed yeast strain were cultivated in SD/-Ura liquid medium (0.67% nitrogen, 0.077% SD/-Ura, 2% glucose, PH = 5.8) to OD_600_ = 1. Each sample of the three yeast strains consisted of 10 mL yeast cell (OD_600_ = 1). The yeast cells in each sample were collected by centrifugation (6,000 g, 5 min), washed with 10 mL sodium citrate buffer (10 mM, pH 4.5) and resuspended in 10 mL transport buffer (10 mM sodium citrate buffer, 2 mM glucose, 20 mM ammonium sulphate, 20 μM ^2^H-IAA, pH 4.5)^25^. ^2^H-IAA (Mr = 177.19) was gifted by Professor Shihong Luo (College of Bioscience and Biotechnology, Shenyang Agricultural University). At four time points (5, 15, 25 and 60 min after sample were resuspended in 10 mL transport buffer), the yeast cells in each sample were collected and washed as above, then were re-collected (6,000 g, 10 min) and the supernatant removed gently. The cell walls of these yeast cells were dissolved in Lysing Enzymes (75 μL yeast lytic enzyme, 15 μL β-mercaptoethanol, 1410 μL sorbitol buffer) (Solarbio, Beijing, China) and placed at 30 °C for 2 h, then stored in -80 °C until determination of ^2^H-IAA concentration. Three independent replicates were used in the uptake test.

The apical buds and Internode 3 were sliced from seedlings of CB*t* and CB*d* (with seven internodes) at the same maturity stage using a razor. Seedlings were grown in greenhouse as above. Three independent samples of each tissue of CB*t* and CB*d* were collected and immersed in liquid nitrogen. The sample was ground into a powder using pestle and mortar. 100 mg of powder from each sample were placed in cryogenic vials.

The ^2^H-IAA uptake by yeast cell and free IAA concentrations in apical buds and Internode 3 were detected and quantified with ultra-high performance liquid chromatography (Shimadzu, LC-20AD, Japan) and QQQ-triple quadrupole mass spectrometer (Shimadzu, LCMS-8040, Japan) (LC–MS) using a positive electrospray ionization (ESI) detection system. 5 ng N_5_-IAA was used as the internal standard and 1 ml ethyl acetate was added to each sample. After vortex for 10 min, the sample was centrifuged (13,000 g, 10 min) and the supernatants transferred to new 2 mL polypropylene tubes. Residual pellets were extracted again but with 0.5 mL of ethyl acetate and the supernatants were combined. Next, the supernatant was evaporated on a vacuum concentrator (Eppendorf) until dry, 0.5 mL of 70% methanol was added to resuspended the residue, samples were centrifuged and the supernatants transferred to glass vials, for analysis by LC–MS using multiple reaction monitoring (MRM) scan with a windows of 50 s and a target scan time of 3 s. The ^2^H-IAA and free IAA concentrations were quantified as 5 ng (the weight of internal standards) multiplied the ratio of their peak area to the peak area of N_5_-IAA.

### Informed consent for publication

Informed consent for publication is obtained from the participant for the identifiable image in Fig. 1A. The authors confirm that the Scientific Reports and Springer Nature Press have unrestricted copyright to publish or re-publish the figure (with the photographic portrait) for scientific and educational purposes.

## Supplementary Information

Supplementary Information 1.

Supplementary Information 2.
