# Supplementary material for: Bulked segregant analysis reveals candidate genes responsible for dwarf formation in woody oilseed crop castor bean
Source: Sci Rep. 2021 Mar 18;11:6277. doi: 10.1038/s41598-021-85644-1 (PMC7973431; doi:10.1038/s41598-021-85644-1)
Supplement: Supplementary file 1 — Supplementary Information 1. [file 41598_2021_85644_MOESM1_ESM.doc]

Bulked segregant analysis reveals candidate genes responsible for dwarf formation in woody oilseed crop castor bean

Zaiqing Wang^1,3^, Anmin Yu^2^, Fei Li^1,3^, Wei Xu^1,3^, Bing Han^1,3^, Xiaomao Cheng^2^, Aizhong Liu^2^,*

^1^ Department of Economic Plants and Biotechnology, Yunnan Key Laboratory for Wild Plant Resources, Kunming Institute of Botany, Chinese Academy of Sciences, Kunming 650204, China;

^2^ Key Laboratory for Forest Resources Conservation and Utilization in the Southwest Mountains of China, Ministry of Education, Southwest Forestry University, Kunming 650224, China;

^3^ University of the Chinese Academy of Sciences, Beijing 100049, China

***** Correspondence: e-mail: liuaizhong@mail.kib.ac.cn (AL)

Tel.: +86-871-6522-3125

Orcid ID : 0000-0002-3197-5535

**Table S1.** Assessment of plant height and related traits between CB*t* and CB*d.*

|  | PH (cm) | VHSB (cm) | HPR(cm) | NN (n) | DMS (cm) | ALI (cm) |
| --- | --- | --- | --- | --- | --- | --- |
| CB*t* | 236.5 ± 19.68 | 103.83 ± 9.81 | 71.83 ± 7.17 | 14 ± 0.52 | 14.33 ± 1.84 | 5.25 ± 0.65 |
| CB*d* | 45.67 ± 23.76 | 13.67 ± 23.67 | 22.67 ± 10.02 | 17 ± 1.00 | 4.76 ± 2.02 | 1.32 ± 0.39 |
| *p* value | 3.91*e*-6 | 6.51*e*-5 | 5.72*e*-5 | 0.018 | 1.72*e*-4 | 1.02*e*-6 |

PH: plant height, VHSB: vertical height of the secondary branching, HPR: height of primary raceme, NN: number of node on main stem, DMS: diameter of main stem, ALI: average length of internodes on main stem. The average values ± SD and *p* value of Student’s *t*-test were calculated based on the data from at least seven independent plants of CB*h* or CB*d*.

**Table S2.** Summary of the sequencing results data

|  | CB*t* | CB*d* | Tall bulk | Dwarf bulk |
| --- | --- | --- | --- | --- |
| Raw data (Gb) | 12.61 Gb | 15.95 Gb | 11.96 Gb | 12.60 Gb |
| HQ clean data (Gb) | 11.02 Gb | 12.80 Gb | 10.45 Gb | 10.89 Gb |
| Q20 (%) | 98.00 % | 98.03 % | 98.02 % | 98.04 % |
| Q30 (%) | 94.59 % | 94.52 % | 94.61 % | 94.65 % |
| Raw reads | 84,099,740 | 106,337,444 | 79,741,716 | 83,983,462 |
| HQ clean reads | 79,990,550 | 97,473,738 | 75,842,138 | 79,979,694 |
| Depth (×) | 34.13 × | 41.65 × | 32.39 × | 34.16 × |
| Align ratio (%) | 92.28 % | 85.80 % | 94.76 % | 94.75 % |

**Table S3.** Ten castor bean varieties were used SNP genotype identification

| Variety | Country | Latitude | Longitude | PH(cm) | Mean of PH(cm) |
| --- | --- | --- | --- | --- | --- |
| K-5-7(T1) | Kenya | -1.41 | 37.65 | 580 | 539.2 ± 31.76 |
| K-3-5(T2) | Kenya | -1.10 | 35.86 | 561 |  |
| K-3-13(T3) | Kenya | -1.10 | 35.84 | 534 |  |
| E-5-3(T4) | Ethiopia | 8.33 | 38.98 | 521 |  |
| K-1-15(T5) | Kenya | -0.28 | 36.89 | 500 |  |
| PI_167288(D1) | Turkey | 39.92 | 32.87 | 171 | 153.4 ± 13.16 |
| PI_241368(D2) | Brazil | -15.82 | -47.84 | 155 |  |
| YT384(D3) | China | 39.81 | 121.04 | 155 |  |
| PI_265509(D4) | Colombia | 4.66 | -74.05 | 152 |  |
| PI_243062(D5) | Brazil | -15.82 | -47.84 | 134 |  |

**Table S4.** The primers were used in present research for different experiments

| Primer | Sequence 5,------ 3, |
| --- | --- |
| RcActin2-S | TTGGAATGGAAGCTGCAGGA |
| RcActin2-A | CCTTGCTCATACGGTCTGCAA |
| 5NG4-1-cDNA F | ATGGCTTCAATATACTGTTACAAG |
| 5NG4-1-cDNA F | TTATGCATCTTCAACTTTTTTGCTT |
| pS1300 5NG4-1-GFP F | CTGCAGGGGCCCGGGGTCGACATGGC  TTCAATATACTGTTACAAG |
| pS1300 5NG4-1-GFP R | CATGGTACCGGATCCACTAGTTGCATC  TTCAACTTTTTTGCTT |
| pDR196 5NG4-1 expression F | AGTGGATCCCCCGGGCTGCAGATGGCT  TCAATATACTGTTACAAG |
| pDR196 5NG4-1 expression R | GGGCCCCCCCTCGAGGTCGACTTATGCA  TCTTCAACTTTTTTGCTT |
| pDR196 5NG4-1-GFP F | AGTGGATCCCCCGGGCTGCAGATGGCTT  CAATATACTGTTACAAG |
| pDR196 5NG4-1-GFP R | GGGCCCCCCC TCGAGGTCGACTTACTTG  TACAGCTCGTCCATG |
| 5NG4-1 RT-PCR-F | TGCACCAGTTTGCTTCCTAGC |
| 5NG4-1 RT-PCR-R | ACATAGACGGGTCCCTTCTGG |
| ScActin F | TGCAAACCGCTGCTCAATCT |
| ScActin R | AGAACCACCAATCCAGACGG |
| 29822.t000050 SNP-F | TCCAGCAGAAATACAAGTGAC |
| 29822.t000050 SNP-R | AGTATGCAAGGGCAGCTAA |
| 29846.t000007 SNP-F | AAACGGGATCCCATTGCT |
| 29846.t000007 SNP-R | TCACAGTTGCATCCGCAG |
| 29846.t000008 SNP-F | ATTGAATTGCCAAGGGTC |
| 29846.t000008 SNP-R | AAACAGCTCGACTATCTCCA |
| 29701.t000013 SNP-F | TAGATGGATGGCAGAACG |
| 29701.t000013 SNP-R | CATCTGAGACATTTGCAGC |
| 28345.t000007 SNP-F | GTGAATAATGCCGGGACA |
| 28345.t000007 SNP-R | GGTAACCTTTAGTTGCTGCAT |
