# Supplementary material for: Bulked segregant analysis reveals candidate genes responsible for dwarf formation in woody oilseed crop castor bean
Source: Sci Rep. 2021 Mar 18;11:6277. doi: 10.1038/s41598-021-85644-1 (PMC7973431; doi:10.1038/s41598-021-85644-1)
Supplement: Supplementary file 2 — Supplementary Information 2. [file 41598_2021_85644_MOESM2_ESM.doc]

Bulked segregant analysis reveals candidate genes responsible for dwarf formation in woody oilseed crop castor bean

Zaiqing Wang^1,3^, Anmin Yu^2^, Fei Li^1,3^, Wei Xu^1,3^, Bing Han^1,3^, Xiaomao Cheng^2^, Aizhong Liu^2^,*

^1^ Department of Economic Plants and Biotechnology, Yunnan Key Laboratory for Wild Plant Resources, Kunming Institute of Botany, Chinese Academy of Sciences, Kunming 650204, China;

^2^ Key Laboratory for Forest Resources Conservation and Utilization in the Southwest Mountains of China, Ministry of Education, Southwest Forestry University, Kunming 650224, China;

^3^ University of the Chinese Academy of Sciences, Beijing 100049, China

***** Correspondence: e-mail: liuaizhong@mail.kib.ac.cn (AL); Tel.: +86-871-6522-3125

Orcid ID : 0000-0002-3197-5535


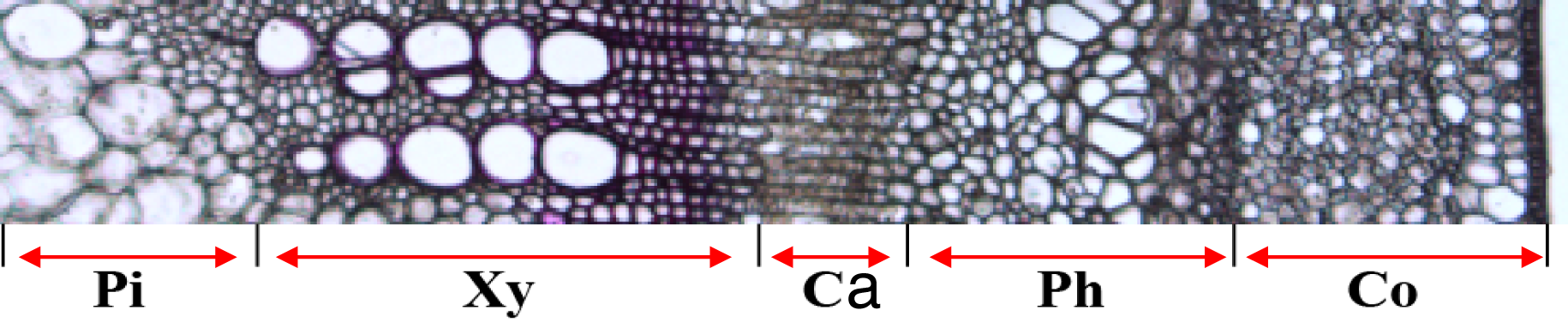


**Figure S1.** Transverse section of Internode 3 at the stage of the seventh internode formation. The sectioned tissue was stained with 1 % saffron and observed under fluorescence microscope.

Pi, Xy, Ca, Ph and Co denote pith, xylem, cambia, phloem and cortex, respectively.


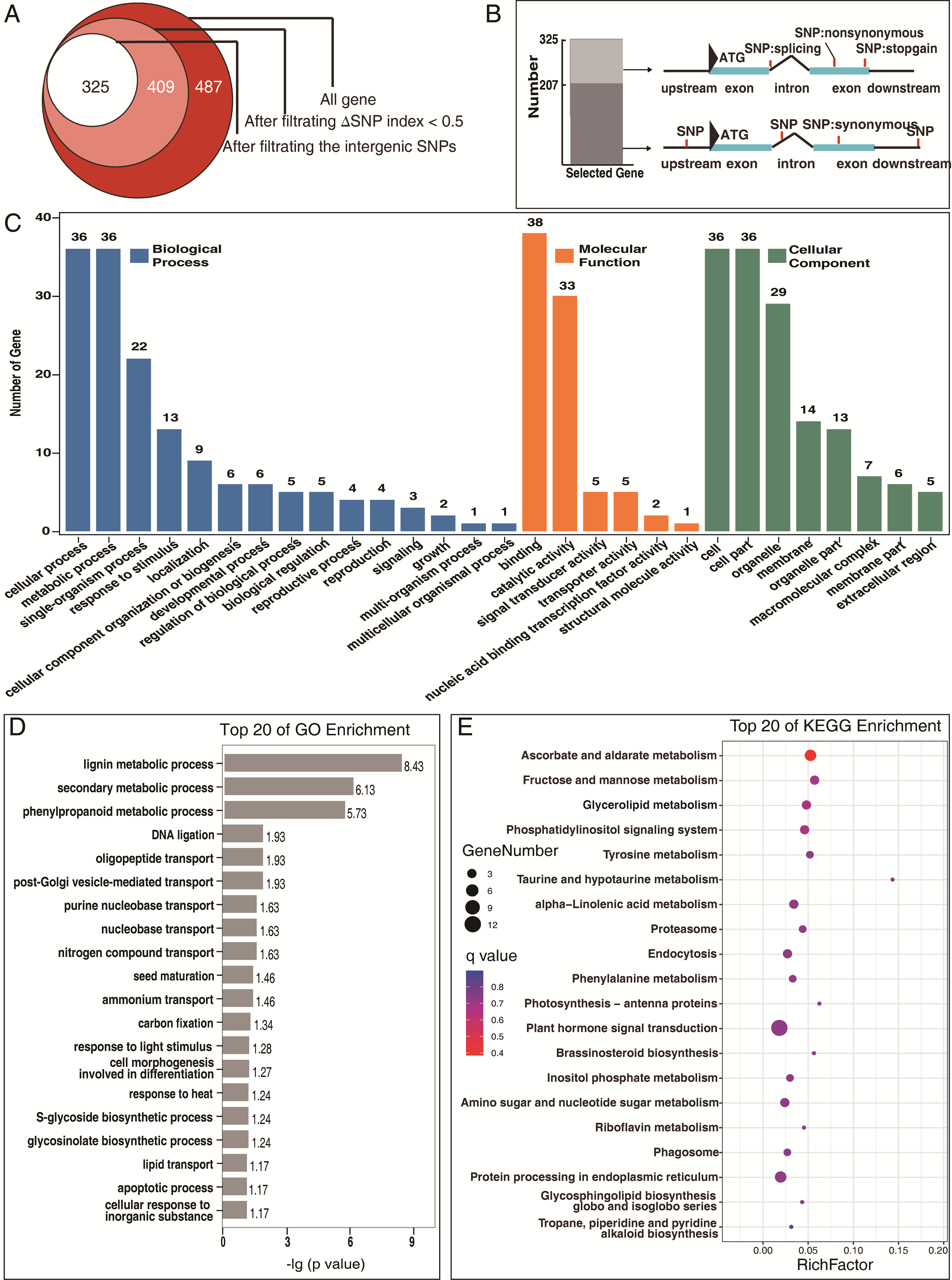
**Figure S2.** Sorting and selection of candidate genes from 487 genes located at *QTL1* and *QTL2*.

(A) Scheme of sorting and selection genes obtained QTL regions. (B) Numbers of candidate genes and positions of SNP occurring on the 325 selected genes. (C) GO enrichment analysis of 325 selected candidate genes based on Biological process (blue bar), Molecular function (orange bar) and Cellular component (green bar), showing the number of genes across the GO terms. (D) The top 20 GO pathways enriched for the 325 candidate genes. The *p* values (hypergeometric test) with -lg transformation were shown. (E) KEGG enrichment analyses of the 325 candidate genes. The top 20 pathways of KEGG enrichments and their pathways involved were showed. The size and color of dots exhibit the number and the ratio (*p* values, a ratio of the number of candidate genes mapped to a certain pathway to the total number of genes mapped to this pathway).


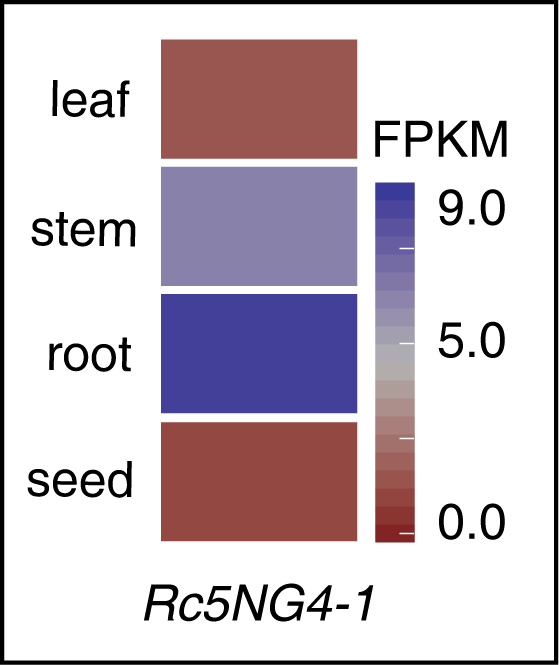


**Figure S3.** A heat map indicating the expression pattern of *Rc5NG4-1* among different tissues (including leaf, stem, root and seed) based on RNA-seq data.

The expression levels of *Rc5NG4-1* among different tissues are represented in fragments per kb per million reads (FPKM). The tissues of leaf, stem and root were obtained from three-week-old seeding, and the seed tissues were obtained after 50 days of pollination. The RNA-seq data were obtained from our castor bean data library (<https://woodyoilplants.iflora.cn/>). The blue indicates the higher expression, and the red shows the lower expression.


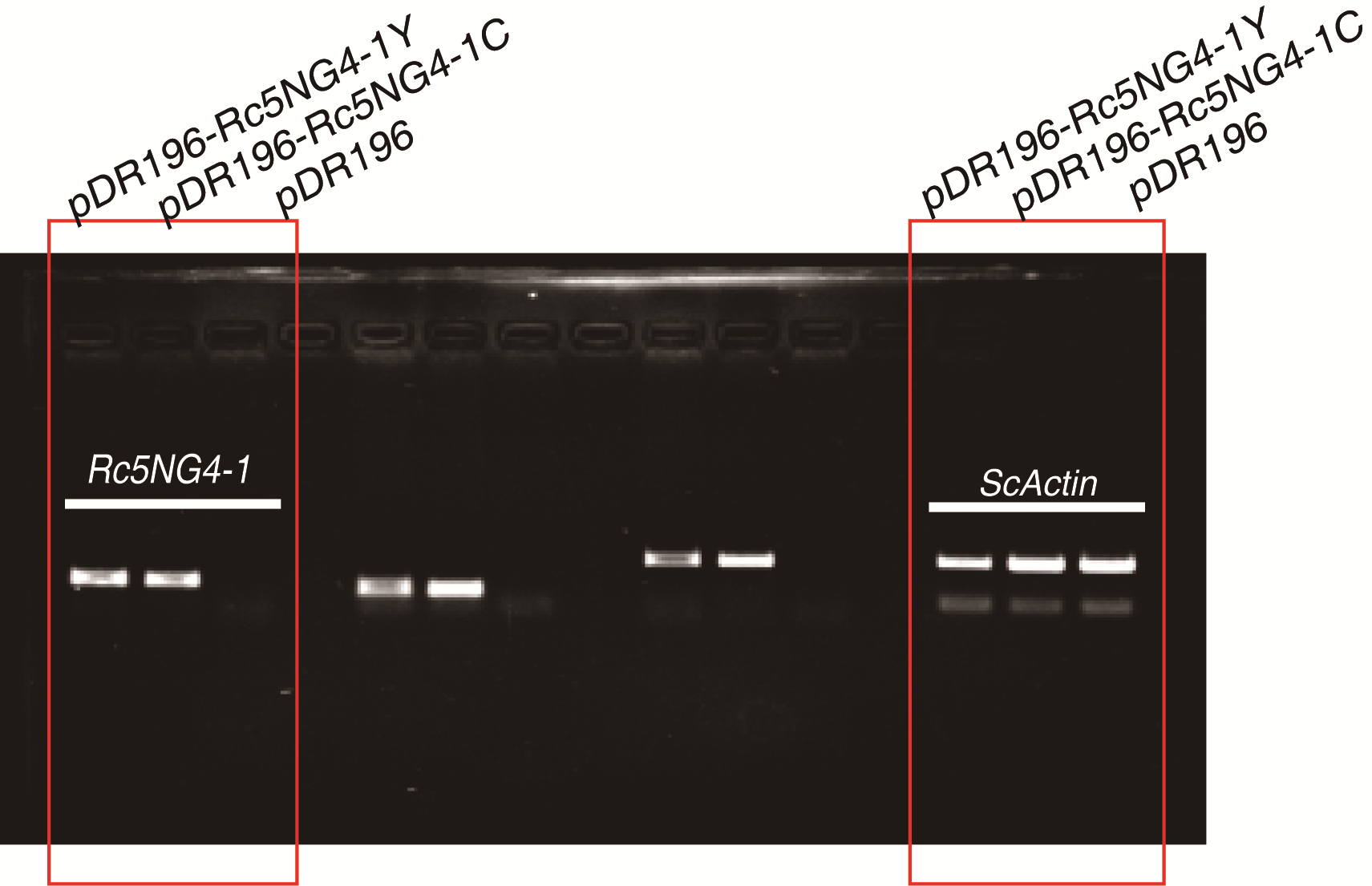


**Figure S4.** Full-length blot for Figure 6B.

pDR196-Rc5NG4-1Y, pDR196-Rc5NG4-1C and pDR196 indicate *Rc5NG4-1Y* transformed yeast, *Rc5NG4-1C* transformed yeast and empty vector pDR196 transformed yeast, respectively. RT-PCR was used to detect the expressional level of 5NG4-1Y and 5NG4-1C in yeast cell, and *ScACTIN* are treated as reference gene.
